# Optimising locational access of deprived populations to farmers’ markets at a national scale: one route to improved fruit and vegetable consumption?

**DOI:** 10.7717/peerj.94

**Published:** 2013-07-02

**Authors:** Amber L. Pearson, Nick Wilson

**Affiliations:** Department of Public Health, University of Otago, Wellington, New Zealand

**Keywords:** Farmers’ market, Fruit and vegetable intake, Geography, Equity

## Abstract

**Background.** Evidence suggests that improved locational access to farmers’ markets increases fruit and vegetable (FV) consumption, particularly for low-income groups. Therefore, we modelled potential alternative distributions of farmers’ markets in one country (New Zealand) to explore the potential impact for deprived populations and an indigenous population (Māori).

**Methods.** Data were collected on current farmers’ markets (*n* = 48), population distributions, area deprivation, and roads. Geographic analyses were performed to optimize market locations for the most deprived populations.

**Results.** We found that, currently, farmers’ markets provided fairly poor access for the total population: 7% within 12.5 km (15 min driving time); 5% within 5 km; and 3% within 2 km. Modelling the optimal distribution of the 48 markets substantially improved access for the most deprived groups: 9% (vs 2% currently) within 12.5 km; 5% (vs 1%) within 5 km; and 3% (vs 1%) within 2 km. Access for Māori also improved: 22% (vs 7%) within 12.5 km; 12% (vs 4%) within 5 km; and 6% (vs 2%) within 2 km. Smaller pro-equity results arose from optimising the locations of the 18 least pro-equity markets or adding 10 new markets.

**Conclusion.** These results highlight the potential for improving farmers’ market locations to increase accessibility for groups with low FV consumption. Given that such markets are easily established and relocated, local governments could consider these results to inform decisions, including subsidies for using government land and facilities. Such results can also inform central governments planning around voucher schemes for such markets and exempting them from taxes (e.g., VAT/GST).

## Introduction

The adequate consumption of fruits and vegetables (FVs) is an important way to prevent a wide range of health problems, including lung cancer (World Cancer Research Fund/American Institute for Cancer Research, 2007), colon cancer (Magalhaes, Peleteiro & Lunet, 2012), breast cancer (Aune et al., 2012), type 2 diabetes (Carter et al., 2010b), stroke (Sherzai et al., 2012), coronary heart disease (Mente et al., 2009), and cognitive decline and dementia (Loef & Walach, 2012). In fact, inadequate FV consumption were ranked as the fifth and 17th highest risk factors for disease, respectively, in the 2010 Global Burden of Disease Study (Lim et al., 2012). Differential intake of FVs by social groups may also contribute to health inequalities (Due et al., 2011; Lakshman et al., 2011; Ministry of Health, 2008).

Given these issues, health authorities recommend increased FV consumption as one of five proposed priority actions to advance non-communicable disease control internationally (Beaglehole et al., 2011). A number of European countries have specifically promoted FVs through food policy and strategic plans, and highlighted the importance of local production for environmental sustainability (DMA, 2009; SNFA, 2009). The latter can potentially involve support for increasing access to farmers’ markets that offer locally grown produce.

Evidence suggests that improved locational access to farmers’ markets can increase FV consumption, even in deprived communities. For example, in the US, a pre-post study without a comparison group showed significant increases in average intake including fruit juice (0.54 servings at pre- to 0.85 at post-intervention), whole fruit (0.49–0.96), green salad (0.44–0.57), tomatoes (0.47–0.67) and other vegetables (0.52–0.75) (Evans et al., 2012).

This effect may partly relate to lower food prices at farmers’ markets, though some specialty markets offer more expensive “organic” produce. But the competitive impact of farmers’ markets might also help drive down FVs prices at neighbouring outlets. For example, one US study found a 12% decrease in supermarket prices for FVs over three years following the introduction of farmers’ markets (Larsen & Gilliland, 2009).

Furthermore, farmers’ market voucher programmes to reduce costs of FVs have been successfully introduced in the US for low-income groups. These vouchers have increased FV intake among low-income recipients (Anderson et al., 2001) and ethnic groups (Racine, Vaughn & Laditka, 2010). Herman et al. (2008) found that farmers’ market vouchers increased daily FV consumption by a significant 1.4 servings compared to the control group and these effects remained after a 6-month follow-up. This increase in consumption even exceeded that found for supermarket vouchers (0.8 servings increase). Another intervention in the US involved establishment of both a voucher scheme and introduction of farmers’ markets in areas with low access to healthy food retailers, low median annual income, and high ethnic diversity. It resulted in over 60% of purchases at the markets being via vouchers, suggesting that improved locational access, paired with improved financial access may be important (Freedman, Bell & Collins, 2011).

Given this background, we aimed to explore the current and potential alternative spatial distributions of farmers’ markets in a national-level modelling study. Our study country, New Zealand, has good national data for the spatial distribution of deprived populations and for Māori (the indigenous population). It is also one where deprived populations have significantly lower FV consumption (Ministry of Health, 2008) and where food insecurity is a problem for low-income populations (Carter, Kruse & Blakely, 2010a; Carter et al., 2011; Morton, Ataoa Carr & Grant, 2012; Parnell et al., 2001; Rush et al., 2007). New Zealand has also had marked growth in the number of farmers’ markets in recent decades and these markets provide potential opportunities for improving access to FV that is locally produced.

## Materials and Methods

### Data

We aimed to identify markets selling locally grown, unprocessed FVs. In New Zealand, membership in Farmers’ Markets NZ Inc indicates that: (1) the market must be a food market; (2) the food production is within a defined local area; and (3) the vendor must be directly involved in the growing/production. We compiled member markets’ addresses and also searched online yellow pages and found 13 additional possible markets. Of these, we verified via telephone interview or online materials that 10 met our inclusion criteria. Thus, we identified a total of 48 qualifying, operating farmers’ markets. We obtained coordinates for their addresses using Google Earth, and imported them into a geographic information system (GIS) for analyses.

Population and area-level deprivation data (NZDep) were compiled at the “meshblock” (MB) level. NZDep consists of nine variables from the 2006 census (Crampton, Salmond & Kirkpatrick, 2004; Salmond, Crampton & Atkinson, 2007). MBs are the finest unit of aggregation in New Zealand (∼41,000 MBs nationally, mean population in 2006 = 195). Population-weighted centroids for census area units (CAUs, the next smallest unit, mean population in 2006 = 2494) were also compiled. Geographic road data were obtained from the Land Information New Zealand 1:50,000 NZTopo database. National FV intake data were derived from the Ministry of Health 2006/7 Health Survey report (Ministry of Health, 2008). These data were used to generate weights for improving FV consumption.

### Location-allocation analyses

Location-allocation models have been for decision-making for health service delivery (Bailey et al., 2011) and facility location. To run these analyses, we first compiled input map data layers: (1) population-weighted centroids of MBs with population and NZDep, used to weight demand of the origin of travel; (2) road junctions; (3) population-weighted centroids of CAUs which represented candidate facility sites; (4) road network arcs; and (5) shortest paths. Data were entered in ArcMap.10 (ESRI, Redlands, CA, USA) to create a network database and we used the location-allocation tool of the Network Analyst extension.

Analysis of this network database generates the shortest path between the demand and candidates using specified weights and connectivity restrictions. We set the maximum travel distance at 12.5 km (about 15 min driving time) and set the objective to maximise attendance. To weight demand points, we used the MB NZDep values and the percentage increase required to attain 100% adequate FV consumption. For example, in the most deprived areas, only 55% of the population consumes the recommended FVs (Ministry of Health, 2008). So, we assigned the most deprived MBs a value of 0.45 and multiplied values by the MB’s population to yield a weight.

We then calculated the proportion of the total population, Māori population and deprivation groups served by existing 48 farmers’ markets, at varying network distances (varying shapes, based on the road network and plausible walking and driving times). To determine whether optimal locations could improve access, we built scenario models to answer: (i) What if this country had no existing farmers’ markets and a total of 48 markets were optimally distributed to meet the dietary needs of deprived populations?; (ii) What if we altered only the existing 18 markets which have the lowest cumulative weighting for the populations served within the 12.5 km buffer (15 min driving time)?; and (iii) What if there were 10 new markets, in addition to the existing 48? Suitable candidate locations (48 sites optimised in Scenario 1, 18 relocated in Scenario 2 and 10 in Scenario 3) were identified and paths were saved for each scenario.

### Calculating geographic access measures to compare results

**Figure 1:**
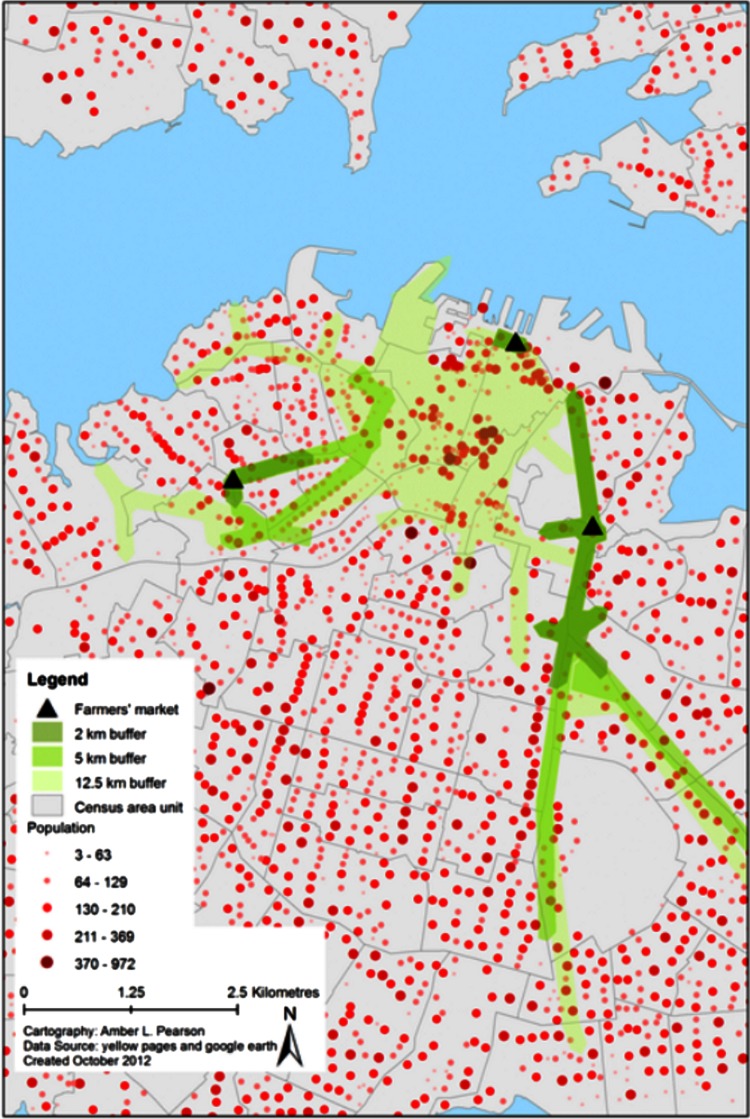
Example of buffering technique showing Auckland City (largest city in New Zealand).

To compare scenarios, we generated measures of population access at varying distances from existing/selected candidate locations, using network buffers. Data on transportation type or distances willing to travel to farmers’ markets in New Zealand do not exist, but US data suggest that 70% of low-income shoppers at farmers’ markets lived within 4 miles (6.5 km), 60% drove and 25% walked to markets (Ruelas et al., 2012). Estimated walking speeds are 4.5–5.5 km/h (kmph) depending on the walker’s age (Carey, 2005) and we used 5 kmph. Speed limits in cities/towns in New Zealand are generally 50 kmph and we used this speed (while recognising congestion, traffic lights and motorway travel may change this). We set buffer distances to approximate the following walking and driving times: (a) 2 km = 24 min walking; (b) 5 km = 1 h walking/6 min driving; and (c) 12.5 km = 15 min driving. Road network buffers were created for each distance from existing/proposed farmers’ markets (Fig. 1). Population, ethnicity and deprivation data for MB population-weighted centroids within buffers were calculated for comparison.

## Results

The current national distribution of farmers’ markets provided fairly poor access for the total population: 7% within 12.5 km (15 min driving time); 5% within 5 km; and 3% within 2 km (Table 1). However, these markets serve a larger proportion of deprived than non-deprived populations.

**Table 1:** New Zealand populations with access to farmers’ markets at various distances under current conditions and three alternative scenarios.

Scenario and demographics	Distance of farmers’ market from residence		
	2 km	5 km	12.5 km
	*(24 min walking)*	*(minutes: 60 walking; 6 driving)*	*(15 min driving)*
**Existing distribution**			
Total population (%)	2.7	4.6	7.1
Total Māori population (%)	2.2	4.3	6.8
Total population, in 30% most deprived areas (%) [D]	0.8	1.4	2.4
Total population, in 30% most advantaged areas (%) [A]	0.5	1.0	1.5
Ratio of [D] to [A]	1.6	1.4	1.6
Those within buffer in 30% most deprived areas (%) [D2]	30.3	29.2	33.6
Those within buffer in 30% most advantaged areas (%) [A2]	19.1	20.8	21.0
Ratio of [D2] to [A2]	1.6	1.4	1.6
**Scenario 1 (all optimised)**			
Total population (%)	7.4	15.7	26.7
Total Māori population (%)	5.7	12.4	21.8
Total population, in 30% most deprived areas (%) [D]	2.7	5.4	8.6
Total population, in 30% most advantaged areas (%) [A]	1.5	3.3	6.5
Ratio of [D] to [A]	1.8	1.6	1.3
Those within buffer in 30% most deprived areas (%) [D2]	36.7	34.5	32.1
Those within buffer in 30% most advantaged areas (%) [A2]	20.7	21.3	24.3
Ratio of [D2] to [A2]	1.8	1.6	1.3
**Scenario 2 (18 relocated)**			
Total population (%)	5.8	12.6	21.5
Total Māori population (%)	3.9	8.7	16.0
Total population, in 30% most deprived areas (%) [D]	1.8	3.5	6.1
Total population, in 30% most advantaged areas (%) [A]	1.3	3.0	5.5
Ratio of [D] to [A]	1.4	1.2	1.1
Those within buffer in 30% most deprived areas (%) [D2]	30.5	28.0	28.5
Those within buffer in 30% most advantaged areas (%) [A2]	21.7	23.7	25.4
Ratio of [D2] to [A2]	1.4	1.2	1.1
**Scenario 3 (10 added)**			
Percent total population (%)	5.4	10.5	16.3
Percent total Māori population (%)	3.7	7.5	12.1
Percent total population, in 30% most deprived areas (%) [D]	1.7	3.1	4.8
Percent total population, in 30% most advantaged areas (%) [A]	0.9	2.1	3.8
Ratio of [D] to [A]	2.0	1.5	1.3
Those within buffer in 30% most deprived areas (%) [D2]	31.7	29.5	29.5
Those within buffer in 30% most advantaged areas (%) [A2]	16.2	20.0	23.2
Ratio of [D2] to [A2]	2.0	1.5	1.3

### Scenario 1: optimised relocation of all 48 market locations

This scenario improved access for deprived groups for all distances: 9% (vs 2% currently) within 12.5 km; 5% (vs 1%) within 5 km; and 3% (vs 1%) within 2 km. We also found improved access for Māori at all distances: 22% (vs 7% currently) within 12.5 km; 12% (vs 4%) within 5 km; and 6% (2%) within 2 km (Table 2). The ratios of high deprivation to low deprivation populations served increased markedly from current conditions to Scenario 1 for the 2 km buffer (from 1.6 to 1.8), and attenuated at greater distances.

**Table 2:** Minimum number of markets at optimised locations nationally to ensure population access within 12.5 km distance of farmers’ market, no weighting (compared to *n* = 48 currently).

Socio-demographic group	Number of markets required to ensure population access within 12.5 km distance of farmer’s market *(15 min driving)*	
	25% have access	50% have access
Total population (all NZ)	16	82
Total Māori population	31	192
Total population, in the most deprived *three* deciles (area deprivation)	15	59

### Scenario 2: relocation of 18 locations (optimised)

When shifting the 18 least pro-equity markets to optimised locations, similar to Scenario 1, we found only slightly less favourable pro-equity results arose relative to Scenario 1: 6% for most deprived three deciles (vs 2% currently) within 12.5 km; 4% (vs 1%) within 5 km; and 2% (vs 1%) within 2 km (Table 1). The ratios of high deprivation to low deprivation populations were the closest to 1.0 (indicating equal access) when compared to existing conditions and every other scenario we evaluated, particularly at the 12.5 km distance.

### Scenario 3: addition of 10 locations

The addition of 10 new markets provided little additional benefit in access for deprived populations, compared to the other scenarios. However, the ratio of high to low deprivation populations served for the 2 km buffer indicated the highest proportion of deprived served, compared to existing conditions and all other scenarios. In addition, there was still some slight improvement over current conditions: 5% (vs 2% currently) within 12.5 km; 3% (vs 1%) within 5 km; and 2% (vs 1%) within 2 km.

**Figure 2:**
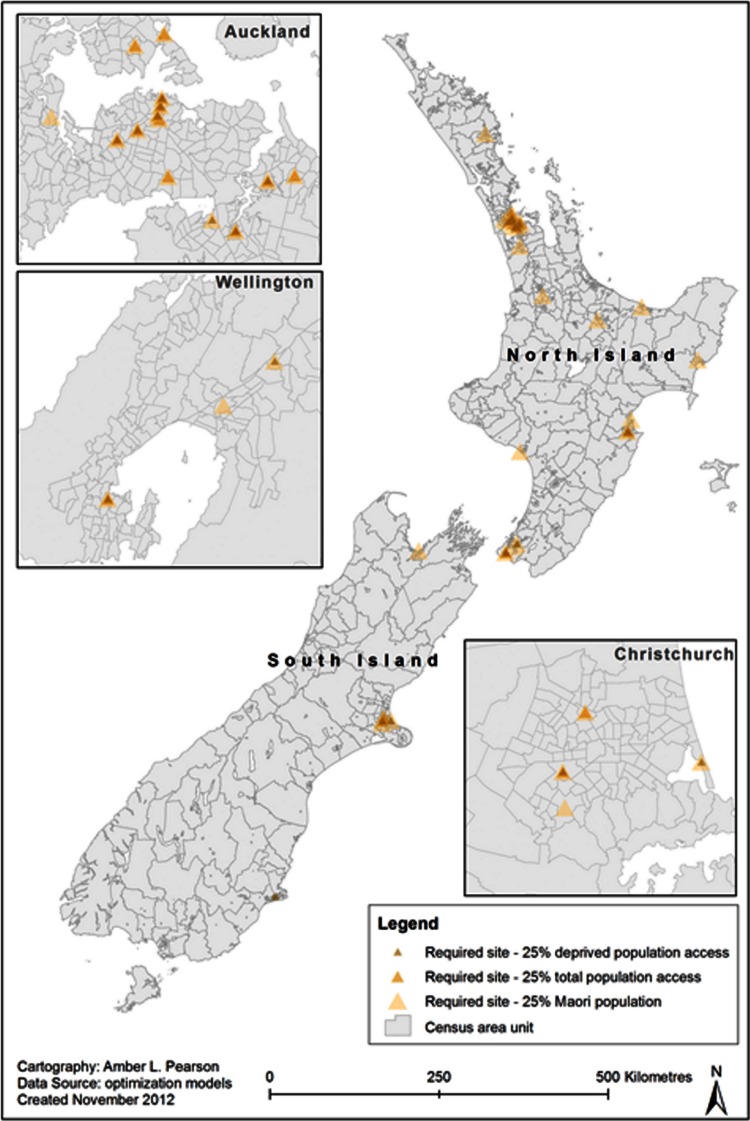
Optimised locations of farmers’ markets to reach 25% of total population, Māori population and deprived groups.

In order to provide 25% of the total population with market access within 12.5 km, 16 markets would be needed at unweighted, optimal locations (Table 2). Similarly, 31 would be needed to provide 25% of the Māori population with access and only 15 needed to provide access to 25% of the most deprived groups (Fig. 2). However, to provide access to 50% of each of these groups, the number of markets increases greatly, to 82, 192, and 59 respectively.

## Discussion

### Interpretation of main findings

In this study it was possible to integrate a large amount of relevant data with GIS software to study optimisation options around improving farmers’ market access. This work was favoured by high quality geographic data on ethnicity and deprivation in the New Zealand setting, but such approaches would seem feasible in many other developed countries.

We found that currently farmers’ market locations serve a relatively small proportion of the population (only 7% within 15 min driving). Nevertheless, for those with access, the market distribution was slightly pro-equity, with better access for more deprived populations for both walking and driving distances. This finding is consistent with research elsewhere indicating that deprived groups have better locational access to farmers’ markets than affluent groups (Widener, Metcalf & Bar-Yam, 2011). However, if markets were more optimally located, then fewer markets would be needed to serve 25% of the deprived population, as some deprived areas are in close proximity to one another and are often densely populated. At some US markets, those earning less than US$15,000/year also constitute the majority of shoppers (55%) (Ruelas et al., 2012).

This modelling work indicated that the distribution of farmers’ markets could be improved for serving deprived groups. Simply by relocating the current number of markets, access for deprived groups increased fourfold (i.e., 9% vs 2% for within 12.5 km). For Māori, the equivalent access improvement was over threefold (22% vs 7%). Hence relocating markets could be a mechanism to reduce health inequalities in New Zealand, a goal of the country’s health authorities. Nevertheless, to more fully improve market access, then larger numbers would be required (as per Table 2).

### Possible policy implications

Given that farmers’ markets are potentially mobile, local governments could consider these results to inform decisions, such as subsidies for using government land and facilities in order to achieve optimal access for particular high need groups. They could also provide direct financial incentives for market relocation and to help advertise the new location to the surrounding communities.

Such results could also inform central governments’ planning around voucher schemes for markets and exempting them from taxes (e.g., GST or VAT). Market voucher schemes would be relevant in countries such as New Zealand where food assistance provided through Social Welfare advances and special needs grants totalled NZ$ 254 million in 2009 (MSD, 2010). Food assistance could serve as an avenue for promotion of FV consumption and could be implemented at either supermarkets and/or farmers’ markets. Some evidence suggests that, among low-income groups, farmers’ market vouchers produce a larger increase in FV consumption than those for supermarkets (Racine, Vaughn & Laditka, 2010). In 1992, the US Congress established the Farmers’ Market Nutrition Program (FMNP) to provide vouchers to low-income mothers and their children (WIC) and the elderly to purchase FVs at farmers’ markets. This programme was paired with a nutrition education programme, whereby FMNP recipients are encouraged to improve and expand their diets by adding fresh FVs, and are educated on how to select, store and prepare FVs (Havas et al., 2003). Coupons redeemed through the FMNP resulted in over US$ 16.4 million in revenue to farmers for the fiscal year 2011 (USDA, 2012). Coupon use and method of benefit payment may be an important consideration, as evidence suggests that the expansion of wireless payment options at US farmers’ markets significantly increased sales to benefit assistance recipients (Bertmann et al., 2012).

## Conclusion

These results highlight the potential utility of GIS modelling in informing changes to the location of farmers’ markets and therefore improving accessibility of fruit and vegetables for deprived populations and an indigenous population. Given that such markets are easy to establish and relocate, there is large scope for local and central governments to promote their optimal location and expansion for reasons of both public health and sustainability.

### Study strengths and limitations

To our knowledge, this is the first study to spatially describe the current distribution of farmers’ markets in a country and also to model optimisation scenarios. It used a wide range of relevant data and used GIS for optimisation analyses. Nevertheless, this study considered the farmers’ markets identified by our criteria and search strategy and so it did not consider other FV markets (e.g., farm stalls). Also this study relied on road networks for determining access and so alternative routes (e.g., cycle paths, shortcuts across parks, foot paths) were not included. These alternative routes could increase access in some areas.

More sophisticated work would also include travel times on public transport networks. It could also consider the location of nearby supermarkets – to determine the potential spatial range of possible competition-induced price reductions for FVs in these supermarkets (as suggested for the US data) (Larsen & Gilliland, 2009).

## Supplemental Information

10.7717/peerj.94/supp-1 Supplemental Information 1 Existing FMs - population access within 2 km

10.7717/peerj.94/supp-2 Supplemental Information 2 Existing FMs - population access within 5 km

10.7717/peerj.94/supp-3 Supplemental Information 3 Existing FMs - population access within 12.5 km

10.7717/peerj.94/supp-4 Supplemental Information 4 Scenario 1 FMs - population access within 2 km

10.7717/peerj.94/supp-5 Supplemental Information 5 Scenario 1 FMs - population access within 5 km

10.7717/peerj.94/supp-6 Supplemental Information 6 Scenario 1 FMs - population access within 12.5 km

10.7717/peerj.94/supp-7 Supplemental Information 7 Scenario 2 FMs - population access within 2 km

10.7717/peerj.94/supp-8 Supplemental Information 8 Scenario 2 FMs - population access within 5 km

10.7717/peerj.94/supp-9 Supplemental Information 9 Scenario 2 FMs - population access within 12.5 km

10.7717/peerj.94/supp-10 Supplemental Information 10 Scenario 3 FMs - population access within 2 km

10.7717/peerj.94/supp-11 Supplemental Information 11 Scenario 3 FMs - population access within 5 km

10.7717/peerj.94/supp-12 Supplemental Information 12 Scenario 3 FMs - population access within 12.5 km
